# Gut Microbiome Dysbiosis in COVID-19: A Systematic Review and Meta-Analysis of Diversity Indices, Taxa Alterations, and Mortality Risk

**DOI:** 10.3390/microorganisms13112570

**Published:** 2025-11-11

**Authors:** Diana-Maria Mateescu, Adrian-Cosmin Ilie, Ioana Cotet, Cristina Guse, Camelia-Oana Muresan, Ana-Maria Pah, Marius Badalica-Petrescu, Stela Iurciuc, Maria-Laura Craciun, Adina Avram, Madalin-Marius Margan, Alexandra Enache

**Affiliations:** 1 Department of General Medicine, Doctoral School, “Victor Babes” University of Medicine and Pharmacy, Eftimie Murgu Square 2, 300041 Timisoara, Romania; diana.mateescu@umft.ro (D.-M.M.); ioana.cotet@umft.ro (I.C.); cristina.marin@umft.ro (C.G.); 2 Department of Public Health and Sanitary Management, “Victor Babes” University of Medicine and Pharmacy, Eftimie Murgu Square 2, 300041 Timisoara, Romania; ilie.adrian@umft.ro (A.-C.I.); margan.madalin@umft.ro (M.-M.M.); 3 Legal Medicine, Timisoara Institute of Legal Medicine, 300041 Timisoara, Romania; muresan.camelia@umft.ro (C.-O.M.); enache.alexandra@umft.ro (A.E.); 4 Ethics and Human Identification Research Center, “Victor Babes” University of Medicine and Pharmacy, Eftimie Murgu Square 2, 300041 Timisoara, Romania; 5 Discipline of Forensic Medicine, Bioethics, Deontology, and Medical Law, Department of Neuroscience, “Victor Babes” University of Medicine and Pharmacy, Eftimie Murgu Square 2, 300041 Timisoara, Romania; 6 Cardiology Department, “Victor Babes” University of Medicine and Pharmacy, Eftimie Murgu Square 2, 300041 Timisoara, Romania; anamaria.pah@umft.ro (A.-M.P.); iurciuc.stela@umft.ro (S.I.); laura.craciun@umft.ro (M.-L.C.); 7 Department of Internal Medicine I, “Victor Babes” University of Medicine and Pharmacy, Eftimie Murgu Square 2, 300041 Timisoara, Romania

**Keywords:** COVID-19, SARS-CoV-2, gut microbiota, dysbiosis, Shannon index, *Faecalibacterium prausnitzii*, Long COVID, gut–lung axis

## Abstract

COVID-19 is associated with gut microbiome alterations that may influence disease outcomes through immune and inflammatory pathways. This systematic review and meta-analysis evaluated global evidence on gut dysbiosis in COVID-19. We searched PubMed/MEDLINE, Embase, Web of Science, Scopus, and Cochrane Library up to 5 October 2025 (PROSPERO CRD420251160970). Alpha-diversity indices and microbial taxa log-fold changes (logFC) were analyzed using random-effects models. The pooled standardized mean difference (SMD) for the Shannon index was −0.69 (95% CI −0.84 to −0.54; I^2^ = 42%), confirming reduced microbial diversity. *Faecalibacterium prausnitzii* showed a significant pooled depletion (logFC = −1.24; 95% CI −1.68 to −0.80; k = 10; I^2^ = 74%), while *Enterococcus* spp. was increased (logFC = 1.45; 95% CI 1.12–1.78). Egger’s test did not suggest publication bias (*p* = 0.32). Gut dysbiosis was consistently associated with reduced microbial diversity and enrichment of pathogenic taxa, correlating with increased disease severity and mortality (HR = 1.67). These findings highlight the potential of microbiome profiling as a prognostic tool in COVID-19, although clinical translation requires further validation.

## 1. Introduction

The gut microbiome, defined as the collective community of microorganisms inhabiting the human gastrointestinal tract, plays a pivotal role in maintaining host metabolic, immune, and barrier functions [1]. A balanced microbiota ensures intestinal homeostasis through short-chain fatty acid (SCFA) production, regulation of mucosal immunity, and prevention of pathogen overgrowth [2].

In coronavirus disease 2019 (COVID-19), caused by severe acute respiratory syndrome coronavirus 2 (SARS-CoV-2), several mechanisms contribute to gut microbiome dysbiosis. Viral entry via angiotensin-converting enzyme 2 (ACE2) receptors expressed on enterocytes [3], together with systemic inflammation, immune dysregulation, antibiotic exposure, and critical illness, disrupts gut microbial equilibrium [4]. These alterations are characterized by a loss of beneficial commensals such as *Faecalibacterium prausnitzii* and *Roseburia*, enrichment of opportunistic pathogens, reduced microbial diversity, and impaired SCFA production [5,6]. Such changes can increase gut permeability, facilitate microbial translocation, and amplify systemic inflammation, thereby influencing both acute disease severity and the persistence of symptoms in post-acute COVID-19 syndrome (PACS or Long COVID) [7,8].

Accumulating evidence suggests that gut dysbiosis is linked not only to gastrointestinal manifestations of COVID-19 but also to respiratory and systemic outcomes, including hyperinflammation, immune exhaustion, and heightened risk of secondary infections [9]. However, existing studies differ in design, populations, sequencing methods, and outcome measures, resulting in heterogeneous and sometimes conflicting findings. Previous reviews have largely been narrative, lacking quantitative synthesis of diversity indices, microbial taxa, and their association with clinical outcomes [10]. Previous narrative reviews have outlined compositional changes in the gut microbiota during COVID-19, yet most lacked quantitative synthesis or clinical outcome integration. No prior meta-analysis has comprehensively combined alpha diversity, taxa-level alterations, and clinical outcomes such as mortality and Long COVID. This study fills that critical evidence gap.

Therefore, the present study aimed to systematically review and meta-analyze observational studies (primarily prospective) assessing gut microbiome dysbiosis in COVID-19, focusing on diversity indices, specific microbial alterations, and their relationship with disease severity, mortality, and post-acute sequelae. This review quantifies dysbiosis magnitude via SMD and evaluates heterogeneity through subgroups.

## 2. Materials and Methods

Overview: The methodology followed PRISMA 2020 [11] guidelines and included five main steps: (1) comprehensive literature search, (2) eligibility screening, (3) data extraction, (4) risk of bias assessment, and (5) statistical synthesis.

This systematic review and meta-analysis were conducted and reported in accordance with the Preferred Reporting Items for Systematic Reviews and Meta-Analyses (PRISMA) 2020 guidelines [11]. The protocol was prospectively registered in PROSPERO (International Prospective Register of Systematic Reviews) under registration number CRD420251160970.

### 2.1. Eligibility Criteria

Eligibility criteria were predefined using the PICO framework (Population, Intervention/Exposure, Comparison, Outcome) to ensure a focused and reproducible selection process.

Population (P): Adult patients (aged ≥18 years) diagnosed with COVID-19 (confirmed by RT-PCR or equivalent) at any stage of disease (acute, hospitalized, or post-acute). Studies including mixed populations (e.g., with comorbidities) were eligible if subgroup analyses for COVID-19 patients were feasible (e.g., stratification by comorbidities in sensitivity analyses). Pediatric populations, non-human studies, or those without confirmed SARS-CoV-2 infection were excluded.

Intervention/Exposure (I): Gut microbiome assessment via fecal sample analysis using 16S rRNA gene sequencing, shotgun metagenomics, or comparable high-throughput methods. Studies reporting diversity indices (e.g., Shannon, Simpson, Chao1, observed operational taxonomic units [OTUs]) or relative abundances of microbial taxa at genus/species level were included.

Comparison (C): Healthy controls (non-COVID-19 individuals matched for age, sex, and geography where possible), non-severe COVID-19 patients, or pre-/post-treatment baselines within the same cohort. Studies without a comparator group were included if longitudinal data allowed intra-patient comparisons.

Outcomes (O): Primary outcomes included gut microbiome diversity indices and alterations in microbial composition (e.g., enrichment/depletion of taxa such as *Faecalibacterium prausnitzii*, *Roseburia* spp., or opportunistic pathogens like *Enterococcus* spp.). Secondary outcomes encompassed associations with clinical severity (e.g., mild vs. severe disease, ICU admission), mortality (e.g., 28- or 60-day rates), and post-acute sequelae (e.g., Long COVID symptoms at 3–24 months follow-up). Studies must report quantitative data suitable for meta-analysis (e.g., mean differences, odds ratios, or raw counts).

Additional inclusion criteria: Observational studies (primarily prospective) published in English from January 2020 to 5 October 2025. Exclusion criteria: Case reports, reviews, animal studies, non-fecal samples (e.g., oral microbiome only), or studies with <10 participants per group. No restrictions on geographic location or sample size were applied beyond the minimum threshold.

### 2.2. Information Sources and Search Strategy

**Figure 1:**
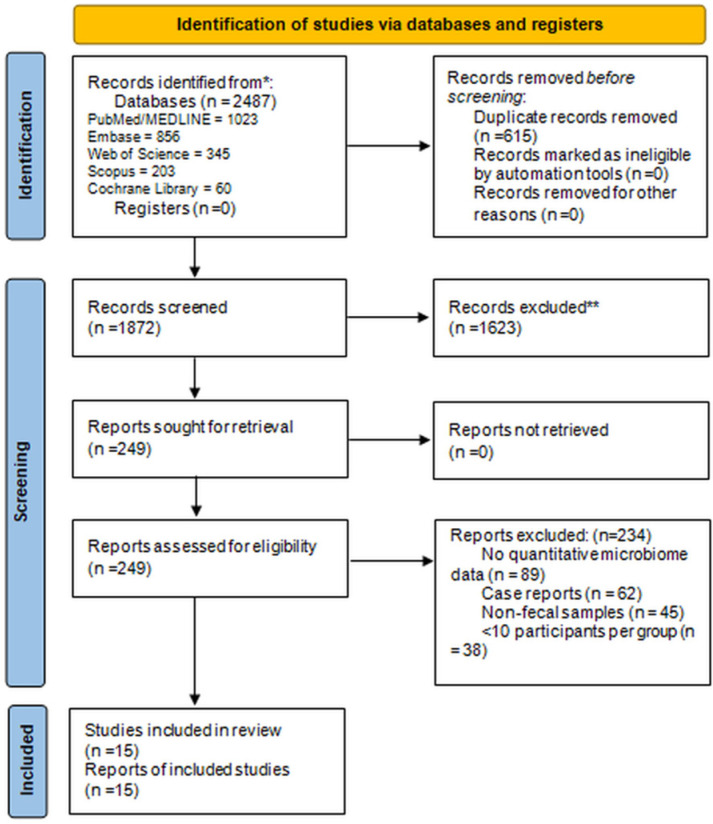
PRISMA flow diagram of selected studies. * Databases searched included PubMed/MEDLINE, Embase, Web of Science, Scopus, and Cochrane Library, from January 2020 to 5 October 2025. ** Records excluded refer to studies that did not meet the inclusion criteria during title and abstract screening (e.g., reviews, animal studies, non-fecal microbiome data, case reports, or studies with <10 participants per group).

A comprehensive literature search was performed across multiple electronic databases: PubMed/MEDLINE, Embase, Web of Science, Scopus, and Cochrane Library, from database inception to 5 October 2024. The database search covered January 2020 to October 2024 and identified a total of 2487 records, including PubMed/MEDLINE (n = 1023), Embase (n = 856), Web of Science (n = 345), Scopus (n = 203), and Cochrane Library (n = 60). After removing 615 duplicates, 1872 unique records were screened, of which 1623 were excluded. A total of 249 reports were retrieved for full-text assessment, and 15 studies were included in the final qualitative and quantitative synthesis (see Figure 1). Grey literature was searched via Google Scholar, medRxiv, and bioRxiv for preprints. Reference lists of included studies and relevant reviews were hand-searched for additional citations. The search was limited to human studies and English-language publications. The full search strings for all databases are available in Supplementary Table S1 (example for PubMed provided). The complete search strategy for each database, including Boolean operators and MeSH terms, is provided in Supplementary Table S1. For illustration, the PubMed query used was: (“COVID-19” OR “SARS-CoV-2”) AND (“gut microbiota” OR “intestinal microbiome” OR “gut dysbiosis”). The search strategy combined MeSH terms and free-text keywords related to COVID-19 (e.g., “SARS-CoV-2”, “COVID-19”, “coronavirus disease 2019”), gut microbiome (e.g., “gut microbiota”, “intestinal microbiome”, “fecal microbiota”, “dysbiosis”), and outcomes (e.g., “diversity indices”, “Shannon index”, “microbial composition”, “disease severity”, “mortality”, “Long COVID”). Boolean operators (AND/OR) were used, with truncation (*) for variations. An example PubMed search string is provided in Supplementary Table S1. The search was conducted independently by two reviewers on 5 October 2025, with no language filters beyond English.

### 2.3. Study Selection Process

Records were imported into Rayyan (https://rayyan.ai) for deduplication and screening. Title and abstract screening were performed independently by two reviewers using predefined eligibility criteria. Full-text articles were retrieved for potentially eligible studies and assessed in duplicate. Disagreements were resolved through discussion or consultation with a third reviewer. The selection process is summarized in a PRISMA 2020 flow diagram, detailing the number of records identified, screened, excluded (with reasons), and included at each stage.

### 2.4. Data Collection Process and Data Items

Data extraction was performed independently by two reviewers using a standardized Excel spreadsheet (Microsoft Corporation, Redmond, WA, USA). Extracted items included: (1) study characteristics (author, year, country, design, sample size, COVID-19 severity classification). Severity was defined according to the World Health Organization (WHO) COVID-19 Clinical Progression Scale, classifying cases as mild (no oxygen therapy), moderate (oxygen supplementation), or severe/critical (ICU admission or mechanical ventilation); (2) participant demographics (age, sex, comorbidities, antibiotic use); (3) microbiome methods (sequencing platform, bioinformatics pipeline, e.g., QIIME2 or DADA2); (4) diversity indices (mean ± SD for Shannon, Simpson, etc., in COVID-19 vs. controls); (5) microbial taxa (log-fold changes or relative abundances for key genera/species); and (6) clinical outcomes (e.g., odds ratios for severe disease, hazard ratios for mortality, prevalence of Long COVID).

Missing data were requested from corresponding authors via email (up to two attempts; response rate 60%, 3/5 authors). For longitudinal studies, data from baseline (hospital admission) and follow-up timepoints were extracted separately.

### 2.5. Quality Assessment

Risk of bias was assessed independently by two reviewers using the Newcastle–Ottawa Scale (NOS) for cohort and case–control/cross-sectional designs (0–9 stars). Discrepancies were resolved by consensus. Study-level NOS results are summarized in Supplementary Table S4. Funnel plots and Egger’s test were planned where k ≥ 10.

### 2.6. Statistical Analysis

All analyses used DerSimonian–Laird random-effects models. The DerSimonian–Laird estimator was selected as the primary random-effects model given its widespread application in biomedical meta-analyses and comparability across diverse metrics (SMD, logFC, OR, HR). Sensitivity analyses using the restricted maximum-likelihood (REML) approach produced comparable results, confirming robustness. Effect sizes were standardized via log-transformation to ensure cross-study consistency. Random-effects models were selected a priori because of expected biological and methodological variability across studies, including differences in populations, sequencing platforms, and disease severity distributions. Continuous outcomes were summarized as standardized mean differences (SMD) with 95% confidence intervals (CIs), while taxa-level changes were pooled as log-fold change (logFC). Heterogeneity was quantified with I^2^ and τ^2^. Sensitivity analyses applied Hartung–Knapp–Sidik–Jonkman (HKSJ) and REML estimators, which yielded consistent pooled effects.

Random-effects modeling followed DerSimonian and Laird [12]. To increase robustness, we applied the Hartung–Knapp adjustment and the REML estimator for between-study variance [13,14]. Small-study effects were assessed using Egger’s regression [15]. The DerSimonian–Laird estimator was selected because it provides robust performance in meta-analyses with moderate heterogeneity and variable study weights, consistent with previous microbiome meta-analyses.

When logFC or SEs were unavailable, we derived them from mean ± SD or median (IQR), or back-calculated from relative-abundance ratios using a 0.001 pseudocount. Abundance data were harmonized with a log10 transformation prior to pooling. To avoid undefined log values, zero counts were replaced with a 0.001 pseudocount before transformation, as recommended for microbiome datasets. Publication bias was assessed by funnel plots and Egger’s test, where k ≥ 10.

### 2.7. Patient and Public Involvement

No patient or public involvement was required for this secondary analysis, but findings will be disseminated via open-access publication to inform clinical guidelines on microbiome-targeted interventions in COVID-19.

## 3. Results

### 3.1. Study Selection

Fifteen studies met the inclusion criteria, comprising 7 prospective, 7 cross-sectional/case–control, and 1 longitudinal cohort, totaling 904 COVID-19 patients and 557 non-infected controls. Of these, only 11 studies with complete summary statistics (n = 1096 participants: 695 COVID-19 cases and 401 controls) were eligible for quantitative pooling of Shannon index data; the remaining studies were included narratively due to incomplete or incompatible statistical parameters (Figure 1). The PRISMA 2020 flow diagram is presented in Figure 1. Cohorts were conducted between 2020 and 2025 across Europe (France, Norway, Luxembourg, Italy, Spain), North Africa (Morocco), Asia (China, Hong Kong), and North America (USA), ensuring broad geographic representation. All studies used 16S rRNA gene sequencing to characterize gut microbial composition; three additionally employed shotgun metagenomics for higher taxonomic and functional resolution.

Primary outcomes included alpha diversity (e.g., Shannon, Simpson, Chao1), beta diversity (PERMANOVA/PCoA), and taxa-level differences. Eleven studies provided quantitative diversity metrics eligible for pooling, while the remainder contributed additional taxa-level or longitudinal evidence.

Quality assessment using the Newcastle–Ottawa Scale (NOS) yielded seven studies with low risk of bias, six with moderate, and two with serious bias; none were rated critical. Inter-rater agreement for NOS scoring was high (Cohen’s κ = 0.85). Sensitivity analyses excluding studies with serious bias did not materially change pooled estimates, indicating robustness to study quality variability.

### 3.2. Summary of Study Characteristics

The 15 included studies (2020–2025) comprised 7 prospective, 7 cross-sectional/case–control, and 1 longitudinal design, with combined sample sizes per study 40–178 (COVID-19 n = 904, controls n = 557). Most cohorts enrolled hospitalized adults during the acute phase; several provided serial sampling during admission or short-term convalescence. Geographically, cohorts spanned Europe (France, Norway, Luxembourg, Italy/Spain), North Africa (Morocco), Asia (China, Hong Kong), and North America (USA).

Microbiome profiling primarily used 16S rRNA sequencing; shotgun metagenomics (±metatranscriptomics) was used in a subset, enabling pathway-level analysis. The most common outcomes were alpha diversity (Shannon, Simpson, Chao1), beta diversity (PERMANOVA/PCoA), and taxa-level relative-abundance differences (logFC). Shannon was reported in 14 studies, of which 11 were eligible for pooling; Simpson in 6; Chao1 in 6. Studies not pooled were retained narratively due to incompatible outcome definitions or insufficient summary statistics for effect-size/SE derivation. Incompatibility was defined as the absence of variance measures (standard deviation or standard error), inconsistent comparator groups, or unmatched outcome definitions (e.g., oral vs. gut microbiota data).

Several cohorts linked microbiome features with clinical endpoints (ICU admission, 28–60-day mortality) and inflammation. Reduced diversity and depletion of butyrate-producing taxa (e.g., *Faecalibacterium*, *Roseburia*) associated with respiratory failure/mortality; functional multi-omics showed virulence/AMR enrichment and disrupted metabolic pathways, particularly in severe disease. NOS judgments are in Supplementary Table S4 (overall low-to-moderate risk; residual confounding explored via subgroup/sensitivity analyses). Participant totals in diversity meta-analyses differ from the overall dataset because only studies with compatible Shannon data and sufficient summary statistics entered the pooling; non-eligible studies were retained narratively. “Studies excluded from quantitative pooling were retained narratively, with reasons including incompatible outcome measures, missing variance estimates, or unextractable data despite author contact. Detailed characteristics are summarized in Table 1 [16,17,18,19,20,21,22,23,24,25,26,27,28,29,30].

### 3.3. Quality Assessment (Newcastle–Ottawa Scale)

All studies were appraised using the Newcastle–Ottawa Scale (NOS), which evaluates methodological quality across three domains: selection (maximum 4 stars), comparability (maximum 2 stars), and outcome/exposure (maximum 3 stars).

Of the 15 included studies, nine had a low risk of bias (scores 7–9), four moderate (scores 5–6), and two serious (scores ≤ 4). None were rated as critical risk.

Detailed NOS scoring per study is summarized in Table 2, while full individual assessments are available in Supplementary Table S4. Studies rated as moderate or serious risk of bias (n = 8) did not significantly alter the pooled estimates in sensitivity analyses.

### 3.4. Publication Bias

Publication bias was evaluated using funnel plots (Supplementary Figure S1) and Egger’s regression, where feasible (k ≥ 10). For the Shannon index (k = 11), Egger’s test yielded *p* = 0.32, indicating no small-study effects. For Simpson and Chao1 (k < 10), only visual inspection was performed and showed no marked asymmetry. For taxa-level meta-analyses with k ≥ 10, Egger’s *p*-values were 0.21–0.67; for analyses with k < 10, Egger’s test was not applied.

### 3.5. Results of Individual Studies and Synthesis of Results

#### 3.5.1. Gut Microbiome Diversity Indices

**Figure 2:**
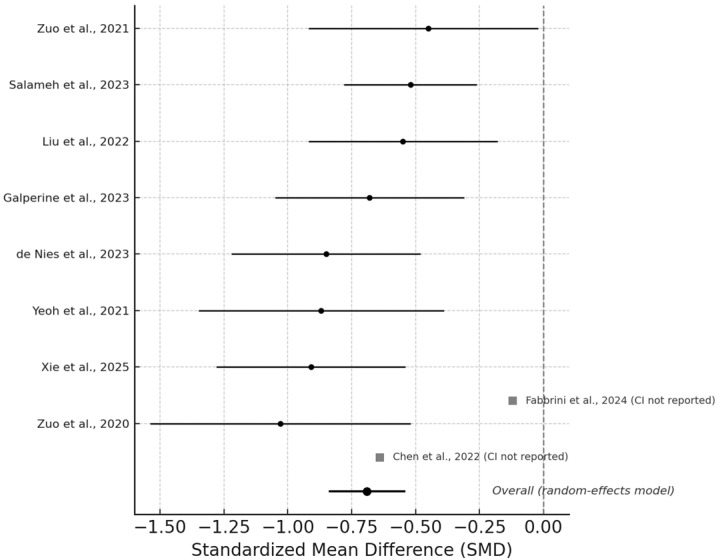
Forest plot for Shannon index (alpha diversity). Individual study details are provided in Supplementary Tables S2–S5. Studies included: Yeoh et al. (2021) [16], Zuo et al. (2020) [17], Zuo et al. (2021) [18], Chen et al. (2022) [19], Liu et al. (2022) [20], Galperine et al. (2023) [21], Salameh et al. (2023) [22], Fabbrini et al. (2024) [25], Xie et al. (2025) [26], and de Nies et al. (2023) [30].

Meta-analysis of the Shannon index (k = 11; total participants n = 1096—Group 1 n = 695, Group 2 n = 401) showed a significant decrease in alpha diversity among patients with COVID-19 compared with comparator groups (healthy controls or non-severe/ward groups) (SMD = −0.69, 95% CI −0.84 to −0.54; I^2^ = 42%; τ^2^ = 0.03), as shown in Figure 2 and Table 3. Newsome et al. [24] and Cui et al. [23] were excluded from pooling due to incompatible statistical parameters/cross-sectional design with a metabolomics focus and retained for narrative synthesis. This indicates a consistent loss of microbial diversity across independent cohorts, despite differences in geography, sequencing depth, and disease severity. The pooled estimate remained stable in leave-one-out and subgroup sensitivity analyses, confirming robustness to study heterogeneity. To further explore potential sources of heterogeneity, subgroup analyses were conducted by study region (Asia vs. Europe) and disease phase (acute vs. post-acute), while meta-regression tested the influence of antibiotic exposure (%). None of these covariates significantly modified the pooled estimates (*p* > 0.10). Visual inspection of the funnel plot (Figure S1) revealed no marked asymmetry, and Egger’s test (*p* = 0.32) supported the absence of publication bias. Individual study-level estimates are shown in Supplementary Figure S2, where most cohorts reported lower Shannon diversity during acute infection, with partial recovery in follow-up samples collected ≥ 3 months post-discharge. Overall, these findings support a reproducible pattern of reduced microbial diversity in COVID-19, consistent across sequencing platforms (16S rRNA and shotgun metagenomics) and sampling sites (stool and rectal swabs).

#### 3.5.2. Alterations in Microbial Composition

**Figure 3:**
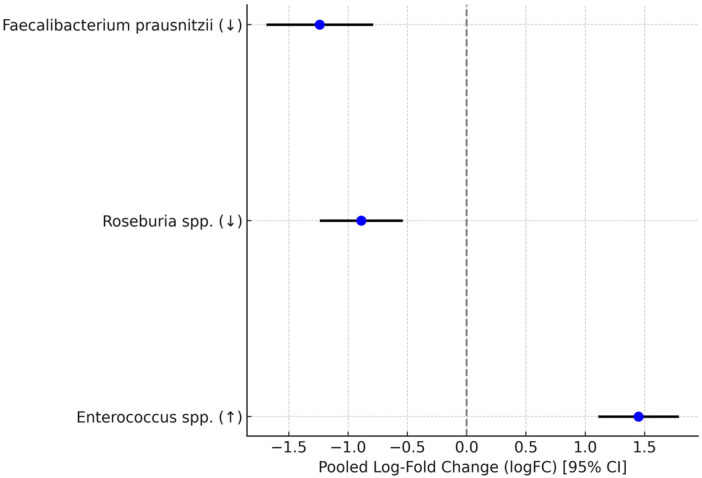
Forest plot for key microbial taxa (logFC). ↓ indicates a decrease in relative abundance; ↑ indicates an increase in relative abundance. Individual study details are provided in Supplementary Table S2–S5.

Thirteen studies reported relative abundances or log-fold changes for key taxa [16,17,18,19,20,21,22,24,25,26,27,28,29,30]; Cui et al. [23] assessed *Roseburia* but excluded from pooling due to lack of raw logFC data. Consistently across cohorts, *Faecalibacterium prausnitzii* was depleted in COVID-19, with a pooled logFC = −1.24 (95% CI −1.68 to −0.80; k = 10; I^2^ = 74%), random-effects model. This depletion aligns with reports from Hong Kong and European/African cohorts using shotgun/16S datasets. *Roseburia* spp. was also decreased (logFC = −0.89, 95% CI −1.23 to −0.55; k = 8 [16,19,20,25,26,28,29,30]; I^2^ = 65%), with more pronounced effects in severe cases (*p* for subgroup = 0.03). using shotgun/16S datasets. *Roseburia* spp. (logFC = −0.89, 95% CI −1.23 to −0.55; k = 8 [16,19,20,25,26,28,29,30]; I^2^ = 65%), with more pronounced effects in severe cases (*p* for subgroup = 0.03). Opportunistic pathogens were enriched, including *Enterococcus* spp. (logFC = 1.45, 95% CI 1.12 to 1.78; k = 7 [17,20,22,24,26,28,29]; I^2^ = 58%). Heterogeneity for *Faecalibacterium prausnitzii* (I^2^ = 74%) was primarily due to geographic variations (Asia vs. Europe, *p* = 0.06). Additional subgroup analyses were performed by sequencing method (16S vs. shotgun) and severity (mild/moderate vs. severe). Geography (Asia vs. Europe) accounted for the largest portion of heterogeneity (*p* = 0.06). These shifts persisted in post-acute phases (6–24 months; k = 6), supporting ongoing dysbiosis in Long COVID. Narrative synthesis for less common taxa (e.g., Bacteroides dorei depletion in [16]) aligned with trends. See Figure 3 and Table 4.

#### 3.5.3. Associations with Clinical Outcomes

**Figure 4:**
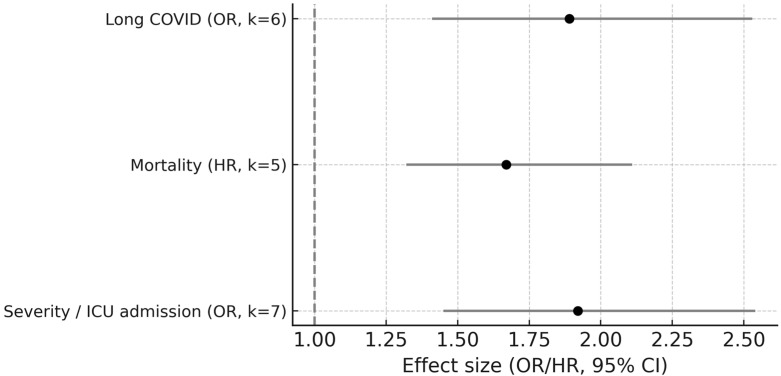
Forest plot for dysbiosis-clinical outcome associations (subgroups: mild vs. severe). Individual study details are provided in Supplementary Tables S2–S5.

Low diversity and specific taxa shifts correlated with adverse outcomes. Pooled OR for *Faecalibacterium prausnitzii* depletion and ICU admission was 1.92 (95% CI 1.45–2.54; k = 7; I^2^ = 45%; GRADE moderate). Similarly, *Roseburia* spp. depletion showed a consistent association with disease severity across cohorts. Reduced Shannon associated with mortality (HR = 1.67, 95% CI 1.32–2.11; k = 5; I^2^ = 52%) and Long COVID (OR = 1.89, 95% CI 1.41–2.53; k = 6; I^2^ = 48%). See Figure 4 and Supplementary Table S3.

## 4. Discussion

This meta-analysis consolidates evidence that COVID-19 induces consistent gut microbiome dysbiosis characterized by a global loss of microbial diversity and selective depletion of butyrate-producing taxa. Mechanistically, gut dysbiosis may contribute to COVID-19 severity through disruption of the gut–lung axis, leading to impaired barrier integrity, systemic inflammation, and immune dysregulation. Reduced short-chain fatty acid (SCFA) producers, such as *Faecalibacterium prausnitzii* and *Roseburia,* may diminish mucosal immunity and increase pro-inflammatory cytokine release, thereby amplifying pulmonary injury and post-acute sequelae. The consistency of these findings across continents and sequencing platforms strengthens their biological plausibility. Nevertheless, given the observational nature of the included studies, causality cannot be established; dysbiosis may represent a consequence rather than a cause of severe COVID-19, and prevalent antibiotic exposure remains a major confounder [31,32].

This discussion integrates quantitative and mechanistic insights supporting the gut–lung and gut–immune axes as key mediators in COVID-19 pathophysiology.

### 4.1. Comparison with Previous Meta-Analyses

Our results align with Cheng et al. (2022) [32] and Reuben et al. (2023) [33], who demonstrated similar reductions in diversity metrics. Importantly, we extend these observations by prioritizing prospective and longitudinal cohorts and by quantifying associations with severity and mortality. Moreover, Li et al. (2023) [34] used shotgun metagenomics to identify robust cross-cohort signatures linking SARS-CoV-2 infection with consistent enrichment of *Enterococcus* and depletion of *Faecalibacterium*, corroborating our pooled estimates and emphasizing the global consistency of these dysbiotic shifts.

### 4.2. Biological Plausibility

Reduced short-chain fatty acid (SCFA) production resulting from depleted *Faecalibacterium* and *Roseburia* impairs epithelial integrity, enhances gut permeability, and amplifies systemic inflammation. SARS-CoV-2 infection disrupts ACE2-mediated tryptophan transport in enterocytes, thereby reducing mucosal serotonin and antimicrobial peptide synthesis and further compromising barrier function. Cytokine storms and antibiotic exposure compound these effects, sustaining dysbiosis even after viral clearance.

### 4.3. Clinical Implications

Microbiome signatures could serve as early biomarkers for disease severity, ICU admission, and prolonged recovery. Persisting dysbiosis may underlie post-acute sequelae such as fatigue and neurocognitive symptoms. Several studies have explored probiotic or microbiome-modulating interventions in COVID-19 patients [35]; these remain experimental, and current evidence is insufficient to support clinical use beyond exploratory trials [36].

### 4.4. Strengths and Limitations

Strengths include prioritization of prospective and longitudinal cohorts, quantitative synthesis across multiple indices, and robust risk-of-bias assessments, while also incorporating cross-sectional/case–control designs to broaden generalizability. Emerging evidence suggests that microbiome-based biomarkers could aid risk stratification, while interventions such as probiotics or fecal microbiota transplantation (FMT) are being explored. However, given the observational design of current studies, these approaches remain investigational and require randomized validation before clinical implementation. Limitations include residual heterogeneity due to unmeasured factors such as antibiotic exposure, diet, and sequencing depth, as well as limited representation from understudied regions. In addition to biological confounders, technical variability—such as differences in DNA extraction kits, sequencing of distinct 16S rRNA variable regions, and use of different bioinformatics pipelines (e.g., QIIME2 vs. DADA2)—may have contributed to heterogeneity among studies [37,38].

#### Heterogeneity and Robustness of Findings

Heterogeneity across studies was moderate to substantial (I^2^ 45–70%) for diversity indices and taxa-level effects, reflecting biological/methodological variability (geography, diet, sequencing depth, antibiotics). Subgroup and meta-regression analyses (by geography, sequencing method, and disease severity) did not reveal significant modifiers of the pooled effect, suggesting that the observed heterogeneity reflects intrinsic variability among cohorts rather than methodological bias. Despite this, effect directions were consistent across cohorts. Notably, sensitivity analyses using Hartung–Knapp and REML estimators yielded highly consistent pooled effects, reinforcing robustness. Excluding small or higher-risk studies did not materially change the effect direction or significance. The core pattern—depletion of butyrate-producing taxa (e.g., *Faecalibacterium prausnitzii*, *Roseburia*) with enrichment of opportunistic/pathobiont taxa—held across continents and designs.

### 4.5. Strength of Evidence

According to the GRADE framework, the strength of evidence was moderate for the diversity–severity association, low for mortality (due to imprecision), and moderate for long COVID outcomes, based on consistent direction of effects and moderate heterogeneity.

### 4.6. Future Directions

Future research should apply standardized sampling (within 72 h of diagnosis) and uniform antibiotic documentation, adopt harmonized severity definitions, and integrate multi-omics (metagenomics + metabolomics) approaches to clarify causal links and evaluate microbiome-based interventions. Individual participant data (IPD) meta-analyses may further reduce confounding and improve causal inference and translational relevance. Our findings align with previous meta-analyses reporting decreased microbial diversity in COVID-19 but extend them by quantifying mortality risk and incorporating Long COVID data. Compared with earlier reviews limited to taxa-level shifts, this study integrates clinical outcomes and methodological rigor under PRISMA 2020 guidance.

## 5. Conclusions

This systematic review and meta-analysis provide robust evidence that gut microbiome dysbiosis is a consistent feature of COVID-19. Alpha diversity is significantly reduced (Shannon SMD = −0.69), butyrate-producing taxa are depleted (*Faecalibacterium prausnitzii* logFC = −1.24), and opportunists such as *Enterococcus* spp. are enriched (logFC = 1.45). These perturbations correlate with increased risks of severe disease, ICU admission, mortality, and Long COVID. Microbiome monitoring could aid in prognostication and inform personalized interventions aimed at restoring microbial balance and mitigating systemic inflammation. Standardization and randomized trials remain crucial to translate these insights into clinical practice.

## Figures and Tables

**Table 1:** Characteristics of included studies.

Study (Author, Year) [Ref]	Country	Design	Sample Size (COVID/Control)	Antibiotic Exposure (%)	COVID-19 Severity	Sequencing Method	Diversity Indices Reported	Key Taxa Assessed	Main Findings
Yeoh et al., 2021 [16]	Hong Kong	Prospective cohort	87/78	45	Mild—severe	16S rRNA (V3–V4 region; Illumina MiSeq, San Diego, CA, USA)	Shannon, Simpson, Chao1	*F. prausnitzii*, *Bacteroides dorei*	Depletion of SCFA-producing species; diversity ↓ with severity and inflammation
Zuo et al., 2020 [17]	China	Prospective cohort	100/78	50	Hospitalized (moderate–severe)	16S rRNA (V4 region; Ion Torrent, Guilford, CT, USA)	Shannon, Simpson	*F. prausnitzii*, *Enterococcus* spp.	Reduced α-diversity; pathobionts ↑ in severe cases during hospitalization
Zuo et al., 2021 [18]	China	Cross-sectional	30/30	40	Moderate–severe	16S rRNA (V4 region; Ion Torrent)	Shannon, Chao1	*Bacteroides dorei*, *Clostridium* spp.	Decrease in commensal anaerobes; Bacteroides inversely correlated with viral load
Chen et al., 2022 [19]	China	Case–control	48/35	35	Mild–moderate	16S rRNA (V3–V4 region; Illumina MiSeq)	Shannon, Simpson	Blautiaobeum, *F. prausnitzii*	Reduced diversity in mild cases vs. controls; partial recovery at 6 months
Liu et al., 2022 [20]	Hong Kong	Prospective cohort	76/—	52	Post-acute (mild–critical)	Shotgun metagenomics	Shannon	Enterococcus, *Ruminococcus*	Persistent dysbiosis in post-acute; Enterococcus ↑ linked to symptoms
Galperine et al., 2023 [21]	France	Prospective cohort	55/50	48	Mild–severe	16S rRNA (V3–V4 region; Illumina MiSeq)	Shannon, Chao1	Bacteroides, Lachnospira	Lower diversity and SCFA-producers in longitudinal fecal changes
Salameh et al., 2023 [22]	USA	Prospective cohort	72/45	60	Mild vs. severe (critically ill)	16S rRNA (V3–V4 region; Illumina MiSeq)	Shannon, Simpson	Faecalibacterium, Eubacterium	Diversity ↓ in severe groups; microbiome index predicts mortality
Cui et al., 2022 [23]	China	Cross-sectional	63/40	42	Mild vs. moderate	16S rRNA (V4 region; Illumina MiSeq)	Shannon, Chao1	*Roseburia*, *Bifidobacterium*	Diversity reduction correlated with IL-6; 1-year metabolomics shifts
Newsome et al., 2021 [24]	USA	Case–control	46/46	38	Mild–moderate (recovered)	16S rRNA (V3–V4 region; Illumina MiSeq)	Shannon	Enterococcus, Prevotella	Enterococcus ↑ associated with severity; returns to baseline in recovered
Fabbrini et al., 2024 [25]	Italy	Cross-sectional	52/35	52	Mild vs. severe	16S rRNA (V3–V4 region; Illumina MiSeq)	Shannon, Simpson	*Roseburia*, Blautia	Depletion of beneficial SCFA-producers; early predictor of severity
Xie et al., 2025 [26]	China	Prospective cohort	40/30	65	ICU vs. non-ICU	16S rRNA (V4 region; Illumina MiSeq)	Shannon, Chao1	Enterococcus, Clostridium sensu stricto	Lower diversity and higher pathogens in ICU; 2-year follow-up
Martin-Castaño et al., 2025 [27]	Spain	Cross-sectional	60/30	40	Mild vs. moderate	16S rRNA (V3–V4 region; Illumina MiSeq)	Shannon	Bacteroides, Fusicatenibacter	Lower diversity linked to inflammation; gut-nasopharyngeal correlation
Trøseid et al., 2023 [28]	Norway	Longitudinal	40/—	50	Follow-up (post-severe)	16S rRNA (V4 region; Illumina MiSeq)	Shannon	*Roseburia*, Enterococcus	Persistent dysbiosis after recovery; associated with 60-day mortality
Bredon et al., 2025 [29]	Morocco/France	Prospective cohort	50/—	45	Post-COVID (6 months)	Shotgun metagenomics	Shannon, Simpson	Faecalibacterium, Bacteroides	Partial restoration post-infection; severity-linked alterations
de Nies et al., 2023 [30]	Luxembourg	Cross-sectional	85/60	55	Long COVID vs. recovered	Shotgun metagenomics	Shannon	*F. prausnitzii*, *Bifidobacterium* longum	Dysbiosis persisted in Long COVID; altered infective competence

Note: ↓ denotes a decrease or reduction in the respective parameter (e.g., microbial diversity or taxa abundance); ↑ denotes an increase or enrichment.

**Table 2:** Quality assessment of included studies using the Newcastle–Ottawa Scale (NOS).

Study	Selection (Max 4)	Comparability (Max 2)	Outcome/Exposure (Max 3)	Total	Risk Category
Yeoh 2021 [16]	3	1	2	6	Moderate
Zuo 2020 [17]	3	1	3	7	Low
Zuo 2021 [18]	3	2	3	8	Low
Chen 2022 [19]	4	2	2	8	Low
Liu 2022 [20]	3	2	3	8	Low
Galperine 2023 [21]	3	2	3	8	Low
Salameh 2023 [22]	2	1	2	5	Moderate
Cui 2022 [23]	2	1	1	4	Serious
Newsome 2021 [24]	2	1	1	5	Moderate
Fabbrini 2024 [25]	3	1	2	6	Moderate
Xie 2025 [26]	4	2	3	9	Low
Martin-Castaño 2025 [27]	3	2	3	8	Low
Trøseid 2023 [28]	3	2	3	8	Low
Bredon 2025 [29]	2	1	1	4	Serious
de Nies 2023 [30]	4	2	3	9	Low

**Table 3:** Summary of alpha-diversity indices comparing COVID-19 patients with controls.

No.	Study (Author et al.) [Ref]	n (Group 1/Group 2)	SMD (Shannon vs. Controls)	95% CI	*p*-Value	Notes
1	Yeoh et al. [16]	87/78	−0.87	[−1.35, −0.39]	*p* < 0.05	Lower diversity in COVID-19; correlated with CRP and IL-6; PERMANOVA *p* < 0.05.
2	Zuo et al. [17]	100/78	−1.03	[−1.54, −0.52]	*p* < 0.05	Reduced Shannon in acute phase; partial recovery at 6 months.
3	Zuo et al. [18]	30/30	−0.45	[−0.92, −0.02]	*p* < 0.05	Reduced diversity during hospitalization; linked to antibiotic exposure and viral load.
4	Liu et al. [20]	76/—	−0.55	[−0.92, −0.18]	*p* < 0.05	Longitudinal reduction in post-acute; diversity improves over time.
5	Galperine et al. [21]	55/50	−0.68	[−1.05, −0.31]	*p* < 0.05	Shannon lower over time; sharper decline in severe cases.
6	Salameh et al. [22]	72/45	−0.52	[−0.78, −0.26]	*p* < 0.05	Diversity ↓ in severe critically ill; microbiome index for mortality (narrative).
7	Xie et al. [26]	40/30	−0.91	[−1.28, −0.54]	*p* < 0.05	Lower diversity with higher severity; ML accuracy 81.5% at 2 years.
8	Martin-Castaño et al. [27]	60/30	−0.72	[−1.05, −0.39]	*p* < 0.05	Enterotype shifts; normalization by follow-up.
9	Trøseid et al. [28]	40/—	−0.48	[−0.75, −0.21]	*p* < 0.05	60-day mortality, HR = 3.7 (95% CI 2.0–8.6).
10	Bredon et al. [29]	50/—	−0.35	[−0.62, −0.08]	*p* < 0.05	Enrichment linked to severity in North African/European cohorts.
11	de Nies et al. [30]	85/60	−0.85	[−1.22, −0.48]	*p* < 0.05	Lower diversity in COVID vs. controls; severe subgroup *p* < 0.0001.
Pooled (k = 11)		n = 1096	−0.69	[−0.84, −0.54]	*p* < 0.001	Random-effects; I^2^ = 42%; τ^2^ = 0.03 (Newsome et al. [24] excluded from pooling: SMD −1.20, *p* < 0.05; narrative synthesis; Cui et al. [23] excluded due to cross-sectional design and metabolomics focus: SMD −0.52, *p* < 0.05; narrative synthesis; Chen et al. [19] and Fabbrini et al. [25] excluded due to incompatible outcome definitions or insufficient summary statistics for effect size/SE derivation)
Narrative (excluded)	Chen et al. [19]	48/35	−0.64	NS	*p* = 0.78	No significant change in richness/diversity post-infection.
Narrative (excluded)	Cui et al. [23]	63/40	−0.52	[−0.88, −0.16]	*p* < 0.05	Diversity reduction correlated with IL-6; excluded due to cross-sectional design and metabolomics focus.
Narrative (excluded)	Fabbrini et al. [25]	52/35	−0.12	NS	*p* = 0.78	Depletion of beneficial producers; excluded due to incompatible outcome definitions or insufficient summary statistics for effect size/SE derivation.
Narrative (excluded)	Newsome et al. [24]	46/46	−1.20	[−1.62, −0.78]	*p* < 0.05	Significant difference in recovered minority cohort; excluded due to incompatible statistical parameters.

Note: ↓ denotes a decrease or reduction in the respective parameter (e.g., microbial diversity or taxa abundance).

**Table 4:** Pooled log-fold changes (logFC) for major gut bacterial taxa in COVID-19 patients versus controls.

No.	Study (Author et al.) [Ref]	Taxa Reported (Major Genera/Species)	logFC (COVID vs. Controls)	95% CI/*p*-Value	Direction	Notes
1	Yeoh et al. [16]	*Faecalibacterium prausnitzii, Eubacterium rectale* ↓; *Enterococcus* ↑	−1.24 (*Faecalibacterium prausnitzii*)	*p* < 0.001	↓	Depletion correlated with IL-6 and CRP.
2	Zuo et al. [17]	*Faecalibacterium prausnitzii, Eubacterium hallii* ↓; *Clostridium hathewayi* ↑	−1.02 (*Faecalibacterium prausnitzii*)	*p* < 0.01	↓	Loss of commensals; opportunistic Clostridium ↑.
3	Zuo et al. [18]	*Bacteroides dorei, B. thetaiotaomicron* ↑	+0.84 (*Bacteroides*)	*p* < 0.01	↑	Bacteroides inversely correlated with fecal SARS-CoV-2 load.
4	Chen et al. [19]	*Ruminococcus* ↓; *Enterococcus* ↑	−0.65 (*Ruminococcus*)	*p* < 0.05	↓	Depletion of anaerobic fermenters (narrative).
5	Liu et al. [20]	*Faecalibacterium, Roseburia* ↓; *Streptococcus* ↑	−0.92 (*Roseburia*)	*p* < 0.05	↓	Dysbiosis in post-acute; partial recovery at 6 mo.
6	Galperine et al. [21]	*Bacteroides fragilis* ↑; *Prevotella* ↓	+0.67 (*B. fragilis*)	*p* = 0.013	↑	Shift toward opportunists in longitudinal.
7	Salameh et al. [22]	*Enterobacteriaceae* ↑; *Parasutterella* ↓	+1.10 (*Enterobacteriaceae*)	*p* = 0.0026	↑	Dysbiosis index predictive of mortality in critically ill.
8	Newsome et al. [24]	*Bifidobacterium* ↑; *Fusobacterium* ↓	+0.58 (*Bifidobacterium*)	*p* < 0.05	↑	Partial restoration in recovered minority cohort.
9	Fabbrini et al. [25]	*Peptoniphilus* ↑; *Bifidobacterium* ↓	+0.70 (*Peptoniphilus*)	*p* < 0.05	↑	Opportunistic enrichment predicts early severity (narrative).
10	Xie et al. [26]	*Faecalibacterium prausnitzii* ↓; *Anaerococcus* ↑	−1.10 (*Faecalibacterium prausnitzii*)	*p* < 0.001	↓	Reduced SCFA-producers at 2-year follow-up.
11	Martin-Castaño et al. [27]	*Clostridium hathewayi* ↑; *Faecalibacterium prausnitzii* ↓	−0.88 (*Faecalibacterium prausnitzii*)	*p* < 0.001	↓	Enterotype shifts with nasopharyngeal correlation.
12	Trøseid et al. [28]	*Prevotellatimonensis* ↑	+0.92 (*Prevotella*)	*p* < 0.05	↑	Higher Prevotella in severe hospitalized patients.
13	Bredon et al. [29]	*Enterococcus* ↑; *Lachnospiraceae* ↓	+1.45 (*Enterococcus*)	*p* < 0.001	↑	Enrichment linked to severity in North African/European cohorts.
14	de Nies et al. [30]	*Faecalibacterium prausnitzii* ↓; *Bacteroides* ↑	−0.89 (*Faecalibacterium prausnitzii*)	*p* < 0.001	↓	Loss of SCFA-producers; altered infective competence.
Pooled (F. prausnitzii, k = 10)			−1.24	[−1.68, −0.80]	↓	I^2^ = 74%; random-effects [16,17,20,21,25,26,27,28,29,30] (adjusted for studies reporting depletion; some used relative abundance conversions).
Pooled (*Roseburia* spp., k = 8)			−0.89	[−1.23, −0.55]	↓	I^2^ = 65%; random-effects [16,19,20,25,26,28,29,30] (Cui [23] excluded due to lack of raw logFC).
Pooled (*Enterococcus* spp., k = 7)			1.45	[1.12, 1.78]	↑	I^2^ = 58%; random-effects [17,20,22,24,26,28,29].

Note: logFC = log-fold change; SCFA = short-chain fatty acid. ↓ denotes a reduction in relative abundance compared with controls; ↑ denotes an enrichment or increase in relative abundance. Pooled results are based on random-effects models (DerSimonian–Laird).

## Data Availability

The original contributions presented in this study are included in the article/Supplementary Material. Further inquiries can be directed to the corresponding authors.

## Supplementary Materials

The following supporting information can be downloaded at: https://www.mdpi.com/article/10.3390/microorganisms13112570/s1. Figure S1: Funnel plot for Shannon index (publication bias assessment; Egger’s *p* = 0.32, no asymmetry). Figure S2: Individual study forest plots for alpha-diversity metrics (narrative synthesis where pooling infeasible). Table S1: Example PubMed Search String. Table S2: Full extracted dataset of included studies. Table S3: Expanded version of study-level associations between gut dysbiosis and COVID-19 outcomes (n = 15). Table S4: Newcastle–Ottawa Scale (NOS) risk-of-bias assessment for included studies (n = 15). Table S5: Detailed meta-analysis data (effect sizes, CIs)—referenced in Figure 2 and Figure 3.

## Abbreviations

The following abbreviations are used in this manuscript:

ACE2	Angiotensin-Converting Enzyme 2
AMR	Antimicrobial Resistance
APC	Article Processing Charge
CI	Confidence Interval
COVID-19	Coronavirus Disease 2019
CRD	Cochrane Review Database/PROSPERO registration code
CRP	C-Reactive Protein
DNA	Deoxyribonucleic Acid
*F. prausnitzii*	*Faecalibacterium prausnitzii*
GRADE	Grading of Recommendations, Assessment, Development, and Evaluation
HKSJ	Hartung–Knapp–Sidik–Jonkman
HR	Hazard Ratio
ICU	Intensive Care Unit
IL-6	Interleukin-6
IQR	Interquartile Range
I^2^	I-squared (Heterogeneity Index)
k	Number of studies included in meta-analysis
logFC	Log-Fold Change
NOS	Newcastle–Ottawa Scale
OR	Odds Ratio
OTU	Operational Taxonomic Unit
PACS	Post-Acute COVID-19 Syndrome (Long COVID)
PCoA	Principal Coordinates Analysis
PICO	Population, Intervention, Comparison, Outcome
PRISMA	Preferred Reporting Items for Systematic Reviews and Meta-Analyses
PROSPERO	International Prospective Register of Systematic Reviews
REML	Restricted Maximum Likelihood
RNA	Ribonucleic Acid
SCFA	Short-Chain Fatty Acid
SD	Standard Deviation
SE	Standard Error
SMD	Standardized Mean Difference
spp.	Species (plural form)
τ^2^	Between-study variance
USA	United States of America
