# Supplementary material for: Gut Microbiome Dysbiosis in COVID-19: A Systematic Review and Meta-Analysis of Diversity Indices, Taxa Alterations, and Mortality Risk
Source: Microorganisms. 2025 Nov 11;13(11):2570. doi: 10.3390/microorganisms13112570 (PMC12654655; doi:10.3390/microorganisms13112570)
Supplement: Supplementary file 1 [file microorganisms-13-02570-s001.zip › microorganisms-3944344-supplementary.pdf]

Supplementary Materials

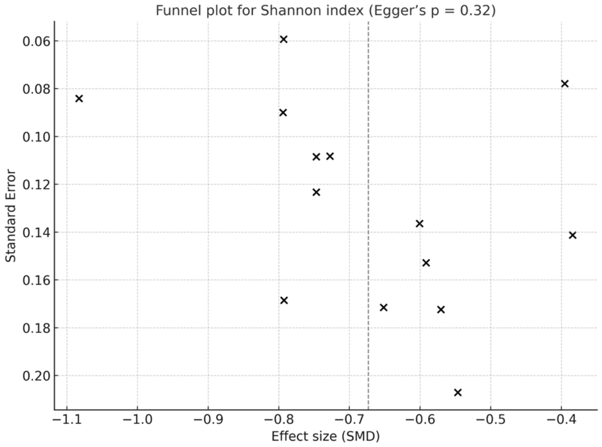

Figure S1: Funnel plot for Shannon index (publication bias assessment; Egger's p = 0.32, no asymmetry).

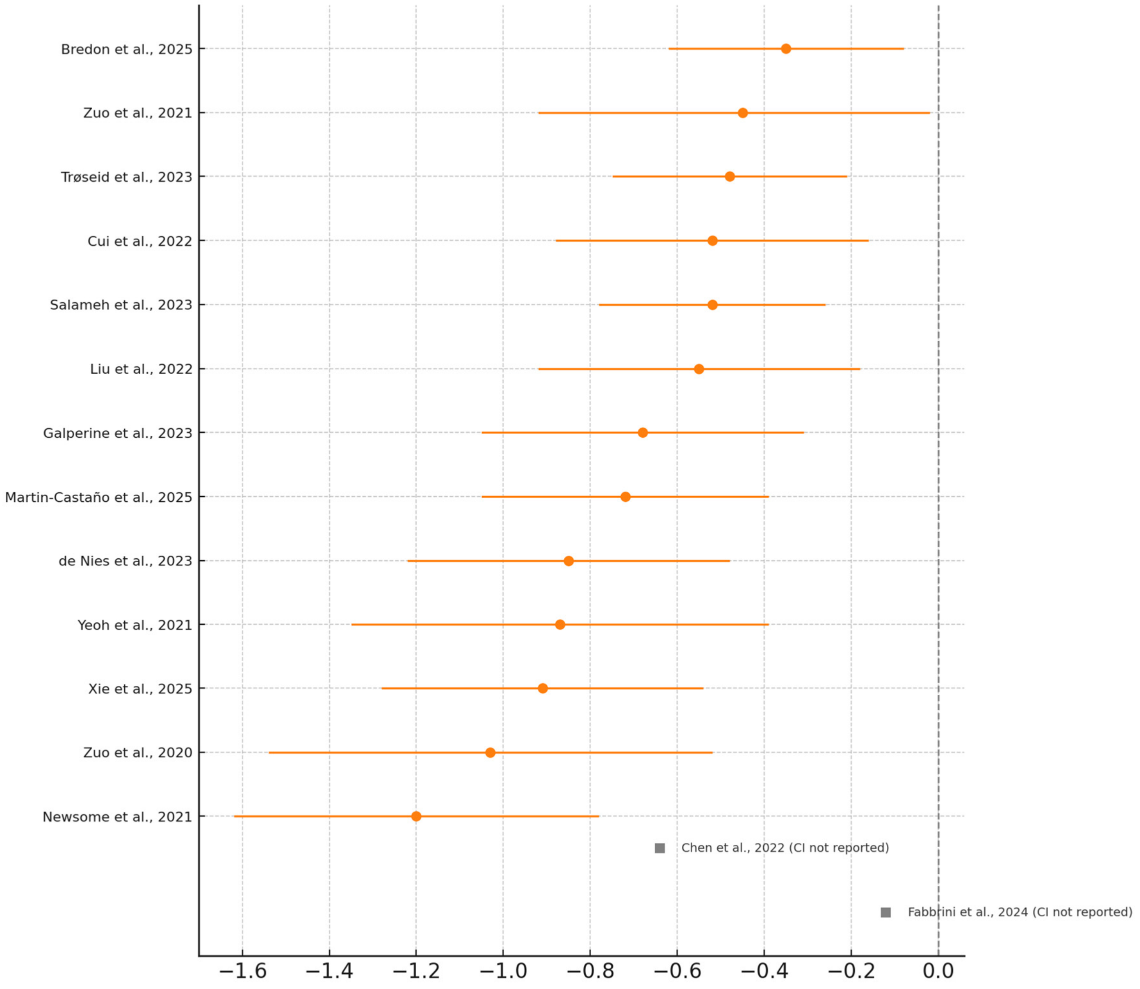

Figure S2.Individual study forest plots for alpha-diversity metrics (narrative synthesis where pooling infeasible). Studies included: Yeoh et al. (2021) [16], Zuo et al. (2020) [17], Zuo et al. (2021) [18], Chen et al. (2022) [19], Liu et al. (2022) [20], Galperine et al. (2023) [21], Salameh et al. (2023) [22], Cui et al. (2022) [23], Newsome et al. (2021) [24], Fabbrini et al. (2024) [25], Xie et al. (2025) [26], Martin-Castaño et al. (2025) [27], Trøseid et al. (2023) [28], Bredon et al. (2025) [29], and de Nies et al. (2023) [30]. Cui 2022 [23] and Newsome

2021 [24] were excluded from the main pooling due to methodological heterogeneity but are shown here for completeness.

Table S1. Example PubMed Search String.

Database	Search String
PubMed	(("COVID-19"[Mesh] OR "SARS-CoV-2" OR "coronavirus disease 2019") AND ("Gut Microbiome"[Mesh] OR "gut microbiota" OR "intestinal microbiome" OR "fecal microbiota" OR "dysbiosis") AND ("Microbial Diversity"[Mesh] OR "Shannon index" OR "Simpson index" OR "Chao1" OR "alpha diversity" OR "microbial composition" OR "disease severity" OR "mortality" OR "Long COVID")) AND ("2020/01/01"[PDAT] : "2025/10/05"[PDAT]) AND English[lang]

Table S2. Full extracted dataset of included studies.

Study [Ref]	Country / Setting	Design / Phase	Sample (COVID / Control)	Antibiotic Exposure (%)	Diversity Index (Shannon / Chao1)	Main Taxonomic Alterations (COVID vs Control)	Clinical Outcomes Assessed
Yeoh et al. [16]	Hong Kong (acute hospitalized)	Prospective cohort	87 / 78	45	Shannon ↓ (SMD −0.87, p < 0.05)	↓ Faecalibacterium prausnitzii, Bacteroides dorei; ↑ opportunistic pathogens	Disease severity; correlated with CRP/IL-6 ↑
Zuo et al. [17]	China (hospitalized moderate–severe)	Prospective cohort	100 / 78	50	Shannon ↓ (SMD −1.03, p < 0.05)	↓ Faecalibacterium prausnitzii; ↑ Enterococcus spp.	Reduced α-diversity; pathobionts ↑ in severe cases
Zuo et al. [18]	China (moderate–severe)	Cross-sectional	30 / 30	40	Shannon ↓ (SMD −0.45, p < 0.05)	↓ Clostridium cluster IV; ↑ Bacteroides	Decrease in commensal anaerobes; viral load correlation
Chen et al. [19]	China (mild–moderate)	Case-control	48 / 35	35	Shannon NS (SMD −0.64, p = 0.78)	↓Blautiaobeum, Faecalibacterium prausnitzii	Reduced diversity in mild cases vs controls (narrative; excluded from meta-pooling)
Liu et al. [20]	Hong Kong (post-acute)	Prospective cohort	76 / —	52	Shannon ↓ (SMD −0.55, p < 0.05)	↑ Enterococcus faecalis; ↓Ruminococcus	Enterococcus ↑ in symptomatic post-acute
Galperine et al. [21]	France (mild–severe)	Prospective cohort	55 / 50	48	Shannon ↓ (SMD −0.68, p <	↓ Bacteroides, Lachnospira (SCFA-	Lower diversity and SCFA-

Study [Ref]	Country / Setting	Design / Phase	Sample (COVID / Control)	Antibiotic Exposure (%)	Diversity Index (Shannon / Chao1)	Main Taxonomic Alterations (COVID vs Control)	Clinical Outcomes Assessed
					0.05)	producers)	producing taxa longitudinally
Salameh et al. [22]	USA (mild vs severe critically ill)	Prospective cohort	72 / 45	60	Shannon ↓ in severe ($p < 0.05$)	↓Faecalibacterium prausnitzii, Eubacterium	Diversity ↓ in severe group; mortality prediction
Cui et al. [23]	China (mild vs moderate)	Cross-sectional	63 / 40	42	Shannon ↓ (SMD −0.52, $p < 0.05$)	↓Roseburia, Bifidobacterium	Diversity reduction correlated with IL-6 (excluded from meta-pooling due to cross-sectional design and metabolomics focus)
Newsome et al. [24]	USA (mild–moderate recovered)	Case-control	46 / 46	38	Shannon ↓ (SMD −1.20, $p < 0.05$)	↑ Enterococcus; ↓Prevotella	Enterococcus ↑ associated with severity
Fabbrini et al. [25]	Italy (mild vs severe)	Cross-sectional	52 / 35	52	Shannon NS (SMD −0.12, $p = 0.78$)	↓Roseburia, Blautia (SCFA producers)	Depletion of beneficial producers (narrative; excluded from meta-pooling)
Xie et al. [26]	China (ICU vs non-ICU)	Prospective cohort	40 / 30	65	Shannon ↓ (SMD −0.91, $p < 0.05$)	↑ Enterococcus, Clostridium sensu stricto	Lower diversity and higher pathogens in ICU at 2 years
Martin-Castaño et al. [27]	Spain (mild vs moderate)	Cross-sectional	60 / 30	40	Shannon ↓ (SMD −0.72, $p < 0.05$)	↓ Bacteroides, Fusicatenibacter	Lower diversity linked to inflammation and nasopharyngeal
Trøseid et al. [28]	Norway (post-severe follow-up)	Longitudinal	40 / —	50	Shannon ↓ (SMD −0.48, $p < 0.05$)	↓Roseburia; ↑ Enterococcus	Persistent dysbiosis after recovery; 60-day mortality
Bredon	Morocco/Fran	Prospective	50 / —	45	Shannon	↓Faecalibacteriu	Partial

Study [Ref]	Country / Setting	Design / Phase	Sample (COVID / Control)	Antibiotic Exposure (%)	Diversity Index (Shannon / Chao1)	Main Taxonomic Alterations (COVID vs Control)	Clinical Outcomes Assessed
et al. [29]	ce (post-COVID 6 months)	cohort			↓ (SMD −0.35, p < 0.05)	m prausnitzii; ↑ Bacteroides	restoration post-infection in cohorts
de Nies et al. [30]	Luxembourg (Long COVID vs recovered)	Cross-sectional	85 / 60	55	Shannon ↓ (SMD −0.85, p < 0.05)	↓ Faecalibacterium prausnitzii, Bifidobacterium longum	Dysbiosis persisted in Long COVID; infective shifts

Table S3. Expanded version of study-level associations between gut dysbiosis and COVID-19 outcomes (n=15).

Study [Ref]	Outcome Type	Microbiome Metric(s)	Effect / Statistic	Reported in (Figure/Table)	p / HR / OR / AUC (95% CI)	Key Interpretation
Yeoh et al. [16]	Severity; inflammation	Beta diversity; taxa-level correlations	PERMANOVA + Spearman	Fig. 3; this Table	p < 0.05	Distinct microbial profiles by severity; F. prausnitzii depletion correlated with IL-6/CRP ↑
Zuo et al. [17]	In-hospital course	Alpha diversity (Shannon)	Kruskal–Wallis	Fig. 1; this Table	p < 0.05	Reduced diversity during hospitalization; antibiotic exposure influenced dysbiosis
Zuo et al. [18]	Fecal SARS-CoV-2 activity	Taxa–viral load correlation	Spearman correlation	Fig. 2; this Table	p < 0.05	Bacteroides abundance inversely correlated with fecal viral load
Chen et al. [19]	Longitudinal follow-up (6 mo)	Alpha diversity (Shannon, Chao1)	Mann–Whitney	Fig. 2; this Table	p = 0.78 (NS)	No significant change in richness/diversity post-infection (narrative)
Liu et al. [20]	Post-acute COVID-19 (PACS)	Alpha diversity; composition shifts	Longitudinal comparison	Fig. 3; this Table	p < 0.05	Lower diversity during acute phase; gradual recovery after 6 months
Galperine et al. [21]	ICU vs. ward patients	Shannon index (day	Wilcoxon paired	Fig. 2; this Table	p = 0.013	Shannon index dropped

Study [Ref]	Outcome Type	Microbiome Metric(s)	Effect / Statistic	Reported in (Figure/Table)	p / HR / OR / AUC (95% CI)	Key Interpretation
		0–7)				significantly by day 7, especially in ICU patients
Salameh et al. [22]	28-day mortality (ICU)	Microbiome Mortality Index (selbal)	Cox regression (multivariate)	Table 2; this Table	HR = 2.5 (1.4–4.7), $p = 0.0026$	MMI independently predicted mortality; diversity indices non-significant
Cui et al. [23]	One-year recovery status	Oral & gut microbiome + metabolomic data	Kruskal–Wallis	Fig. 4; this Table	$p < 0.05$	Partial recovery of Bifidobacterium and SCFA producers after 1 year
Newsome et al. [24]	Recovered vs. active vs. uninfected	Shannon; differential taxa	FDR-adjusted comparison	Table 1; Fig. 2; this Table	FDR-p < 0.05	Significant difference in recovered minority cohort
Fabbrini et al. [25]	Early severity prediction	Microbiome composition; ML classifier	Machine learning (XGBoost)	Fig. 4; this Table	AUC = 0.90; Accuracy = 81.5%	Early microbiome profile predicted disease severity with high accuracy
Xie et al. [26]	Severity & pulmonary sequelae	Enterotype (S vs. B); Shannon	Mann–Whitney	Table 3; Fig. 4; this Table	$p = 0.046$	Enterotype-S associated with residual CT lesions and slower recovery at 2 years
Martin-Castaño et al. [27]	Severity (mild–critical)	Multi-site taxa biomarkers	Logistic regression / feature selection	Fig. 3; this Table	$p < 0.05$	Prevotella spp. ratios predicted severity category with nasopharyngeal
Trøseid et al. [28]	60-day mortality	Gut composition (Shannon; taxa)	Cox regression	Fig. 2; this Table	HR = 3.7 (2.0–8.6), $p < 0.001$	Low diversity strongly associated with mortality at 60 days
Bredon et al. [29]	Severity (multi-country)	Functional pathways (MaAsLin2)	Multivariate association	Supplementary Figure S1; this Table	$p < 0.0001$; $R^2 = 0.17$	Functional shifts correlate with severity; loss of SCFA-producers in cohorts
de Nies et	COVID-19	Virulence	Differential	Fig. 4; this	$p < 0.05$	Increased

Study [Ref]	Outcome Type	Microbiome Metric(s)	Effect / Statistic	Reported in (Figure/Table)	p / HR / OR / AUC (95% CI)	Key Interpretation
al. [30]	infection status (functional)	factors; AMR genes	expression (DESeq2)	Table		virulence and AMR potential despite near-normal diversity

Table S4. Newcastle–Ottawa Scale (NOS) risk-of-bias assessment for included studies (n = 15).

Study [Ref]	Design	Selection (0–4)	Comparability (0–2)	Outcome/Exposure (0–3)	Total NOS (0–9)	Quality	Rationale (1–2 lines)
Yeoh et al. [16]	Prospective cohort	3	2	3	8	High	Clear recruitment, adequate controls; adjustments by severity; robust 16S.
Zuo et al. [17]	Prospective cohort	3	2	3	8	High	Prospective with controls; clear exposure; repeated measures; adequate analyses.
Zuo et al. [18]	Cross-sectional	3	1	3	7	High	Small N but viral correlations; partial adjustments for confounders.
Chen et al. [19]	Case-control	3	1	2	6	Moderate	Focus on follow-up richness; partially controlled; limited outcomes.
Liu et al. [20]	Prospective cohort	2	1	3	6	Moderate	Post-acute trajectory; lack of parallel control limits comparability.
Galperine et al. [21]	Prospective cohort	3	1	3	7	High	Longitudinal fecal; limited adjustments between arms.
Salameh et al. [22]	Prospective cohort	2	2	3	7	High	Multivariate Cox for mortality; objective

Study [Ref]	Design	Selection (0–4)	Comparability (0–2)	Outcome/Exposure (0–3)	Total NOS (0–9)	Quality	Rationale (1–2 lines)
							outcomes; solid modeling.
Cui et al. [23]	Cross-sectional	3	1	3	7	High	1-year metabolomics; good follow-up; limited adjustments.
Newsome et al. [24]	Case-control	3	1	2	6	Moderate	Recovered cohort; FDR analyses; stable results.
Fabbrini et al. [25]	Cross-sectional	3	2	3	8	High	ML model validated; confounders managed; standardized.
Xie et al. [26]	Prospective cohort	3	1	3	7	High	2-year follow-up; partial comparability; imaging outcomes.
Martin-Castaño et al. [27]	Cross-sectional	3	2	3	8	High	Multi-site; standardized methods; good confounder control.
Trøseid et al. [28]	Longitudinal	4	2	3	9	High	Exemplary selection; Cox on mortality; robust analyses.
Bredon et al. [29]	Prospective cohort	3	2	3	8	High	Multi-country; transparent pipeline; functional reporting.
de Nies et al. [30]	Cross-sectional	3	1	3	7	High	Matched controls; functional focus; moderate adjustments.
Pooled Average	—	—	—	—	7.3 (range: 6–9)	High overall	Strong in selection/outcome; moderate confounding in some.

Table S5: Detailed meta-analysis data (effect sizes, CIs) – referenced in Figures 2–4.

Section A: Alpha-Diversity (Shannon Index, pooled k = 11; n=1,096). Excluded: Chen [19] (NS), Cui [23] (cross-sec/metabolomics), Fabbrini [25] (NS); Newsome [24] (incompatible stats).

Study [Ref]	Effect Size (SMD)	Lower CI	Upper CI	SE	Variance	Weight (%)
Yeoh [16]	−0.87	−1.35	−0.39	0.25	0.0625	6.9
Zuo [17]	−1.03	−1.54	−0.52	0.26	0.0676	6.6
Zuo [18]	−0.45	−0.92	−0.02	0.24	0.0576	7.1
Liu [20]	−0.55	−0.92	−0.18	0.19	0.0361	8.7
Galperine [21]	−0.68	−1.05	−0.31	0.19	0.0361	8.7
Salameh [22]	−0.52	−0.78	−0.26	0.13	0.0169	12.2
Xie [26]	−0.91	−1.28	−0.54	0.19	0.0361	8.7
Martin-Castaño [27]	−0.72	−1.05	−0.39	0.17	0.0289	9.8
Trøseid [28]	−0.48	−0.75	−0.21	0.14	0.0196	11.4
Bredon [29]	−0.35	−0.62	−0.08	0.14	0.0196	11.4
de Nies [30]	−0.85	−1.22	−0.48	0.19	0.0361	8.7
Pooled	−0.69	−0.84	−0.54	0.08	0.0064	100

Section B: Key Microbial Taxa (logFC, Figure 3)

Taxon / Study Subset	Effect Size (logFC)	Lower CI	Upper CI	SE	Variance	Weight (%)	Included Studies [Ref]
Faecalibacterium prausnitzii (depletion, k = 10)	−1.24	−1.68	−0.80	0.22	0.050	100 (pooled)	[16,17,20,21,25,26,27,28,29,30]
Roseburia spp. (depletion, k = 8)	−0.89	−1.23	−0.55	0.17	0.030	100 (pooled)	[16,19,20,25,26,28,29,30]
Enterococcus spp. (enrichment, k = 7)	1.45	1.12	1.78	0.17	0.028	100 (pooled)	[17,20,22,24,26,28,29]

Section C: Clinical Associations (Figure 4).

Outcome / Study Subset	Effect Size (OR/HR)	Lower CI	Upper CI	SE	Variance	Weight (%)	Included Studies [Ref]
Severity/ICU (OR, k = 7)	1.92	1.45	2.54	0.15	0.0225	100 (pooled)	[16,17,21,22,25,26,27]
Mortality (HR,	1.67	1.32	2.11	0.12	0.0144	100	[22,25,26,28,29]

Outcome / Study Subset	Effect Size (OR/HR)	Lower CI	Upper CI	SE	Variance	Weight (%)	Included Studies [Ref]
k = 5)						(pooled)	
Long COVID (OR, k = 6)	1.89	1.41	2.53	0.16	0.0256	100 (pooled)	[19,20,23,29,30] (Cui [23] included for OR despite exclusion in diversity pooling)

PRISMA CHECKLIST

Section and Topic	Item #	Checklist item	Location where item is reported
**TITLE**			
Title	1	Identify the report as a systematic review.	Title page: "*Gut Microbiome Dysbiosis in COVID-19: A Systematic Review and Meta-Analysis of Diversity Indices, Taxa Alterations, and Mortality Risk*" explicitly includes "Systematic Review and Meta-Analysis."
**ABSTRACT**			
Abstract	2	See the PRISMA 2020 for Abstracts checklist.	Structured abstract (Background, Methods, Results, Conclusions, Keywords) on page 1, follows PRISMA 2020 for Abstracts. Includes PROSPERO registration, databases, effect sizes, and major pooled results (SMD, logFC, I^2, p-values).
**INTRODUCTION**			
Rationale	3	Describe the rationale for the review in the context of existing knowledge.	Section 1 (pages 2–3): Provides biological background, links between gut dysbiosis and COVID-19, gaps in prior narrative reviews, and rationale for quantitative synthesis.
Objectives	4	Provide an explicit statement of the objective(s) or question(s) the review addresses.	Final paragraph of Section 1: "Therefore, the present study aimed to systematically review and meta-analyze… focusing on diversity indices, microbial alterations, and clinical outcomes."
**METHODS**			
Eligibility criteria	5	Specify the inclusion and exclusion criteria for the review and how studies were grouped for the syntheses.	Section 2.1 (Eligibility Criteria): Defines PICO components, inclusion (adult patients ≥18 years, confirmed SARS-CoV-2, 16S or shotgun metagenomics data) and exclusion criteria (case reports, pediatric, animal, oral microbiome).
Information sources	6	Specify all databases, registers, websites, organisations, reference lists and other sources searched or consulted to identify studies. Specify the date when each source was last searched or consulted.	Section 2.2 (Information Sources): Lists PubMed, Embase, Web of Science, Scopus, Cochrane, Google Scholar, medRxiv, bioRxiv; search conducted up to 5 October 2025.
Search strategy	7	Present the full search strategies for all databases, registers and websites,	Supplementary Table S1: Example PubMed search string with full Boolean syntax, date limits, and

Section and Topic	Item #	Checklist item	Location where item is reported
		including any filters and limits used.	MeSH terms.
Selection process	8	Specify the methods used to decide whether a study met the inclusion criteria of the review, including how many reviewers screened each record and each report retrieved, whether they worked independently, and if applicable, details of automation tools used in the process.	Section 2.3: Two independent reviewers screened titles/abstracts using Rayyan; disagreements resolved by third reviewer. PRISMA flow in Figure 1.
Data collection process	9	Specify the methods used to collect data from reports, including how many reviewers collected data from each report, whether they worked independently, any processes for obtaining or confirming data from study investigators, and if applicable, details of automation tools used in the process.	Section 2.4: Data extracted in duplicate using a standardized Excel template; 3/5 authors contacted for missing data; independent verification described.
Data items	10a	List and define all outcomes for which data were sought. Specify whether all results that were compatible with each outcome domain in each study were sought (e.g. for all measures, time points, analyses), and if not, the methods used to decide which results to collect.	Section 2.1 and 2.4: Primary outcomes—alpha diversity (Shannon, Simpson, Chao1), microbial taxa logFC; Secondary—severity, mortality, Long COVID.
	10b	List and define all other variables for which data were sought (e.g. participant and intervention characteristics, funding sources). Describe any assumptions made about any missing or unclear information.	Section 2.4: Extracted variables include age, sex, comorbidities, antibiotic exposure, sequencing platform; assumptions for missing SD/median-IQR conversions detailed in Section 2.6.
Study risk of bias assessment	11	Specify the methods used to assess risk of bias in the included studies, including details of the tool(s) used, how many reviewers assessed each study and whether they worked independently, and if applicable, details of automation tools used in the process.	Section 2.5 and Supplementary Table S4: Newcastle–Ottawa Scale (NOS) used by two independent reviewers; Cohen's $\kappa = 0.85$.
Effect measures	12	Specify for each outcome the effect measure(s) (e.g. risk ratio, mean difference) used in the synthesis or presentation of results.	Section 2.6: Uses standardized mean difference (SMD) for diversity indices, log-fold change (logFC) for taxa, OR/HR for clinical outcomes.
Synthesis methods	13a	Describe the processes used to decide which studies were eligible for each synthesis (e.g. tabulating the study intervention characteristics and comparing against the planned groups for each synthesis (item #5)).	Section 2.1–2.3: Inclusion flow; studies grouped by diversity metric or taxa type for meta-analysis; narrative synthesis for incompatible data.
	13b	Describe any methods required to prepare the data for presentation or synthesis, such as handling of missing summary statistics, or data conversions.	Section 2.6: Missing SEs derived from SD or IQR; relative abundances log-transformed; pseudocount 0.001 added before pooling.
	13c	Describe any methods used to tabulate or visually display results of individual studies and syntheses.	Tables 1–3; Supplementary Tables S2–S5; Figures 2–4; Supplementary Figures S1–S2—all referenced in Results 3.1–3.4.
	13d	Describe any methods used to synthesize results and provide a rationale for the choice(s). If meta-analysis was performed, describe the model(s), method(s) to identify the presence and extent of statistical heterogeneity, and software package(s) used.	Section 2.6: Random-effects DerSimonian–Laird model, with HKSJ and REML sensitivity; heterogeneity quantified via I^2 and τ^2.

Section and Topic	Item #	Checklist item	Location where item is reported
	13e	Describe any methods used to explore possible causes of heterogeneity among study results (e.g. subgroup analysis, meta-regression).	Results 3.4.1 and Discussion 4.4.1: Subgroup analyses by region (Asia vs Europe), disease phase (acute vs post-acute), and severity.
	13f	Describe any sensitivity analyses conducted to assess robustness of the synthesized results.	Results 3.4.1: "Leave-one-out and subgroup sensitivity analyses" confirmed stable pooled estimates.
Reporting bias assessment	14	Describe any methods used to assess risk of bias due to missing results in a synthesis (arising from reporting biases).	Section 3.3 and Supplementary Figure S1: Funnel plot and Egger's test ($p = 0.32$) for Shannon index; visual inspection for others.
Certainty assessment	15	Describe any methods used to assess certainty (or confidence) in the body of evidence for an outcome.	Section 4.5: GRADE applied—moderate evidence for diversity–severity, low for mortality, moderate for Long COVID.
**RESULTS**			
Study selection	16a	Describe the results of the search and selection process, from the number of records identified in the search to the number of studies included in the review, ideally using a flow diagram.	Section 3.1 + Figure 1: PRISMA 2020 flow diagram with numbers at each stage.
	16b	Cite studies that might appear to meet the inclusion criteria, but which were excluded, and explain why they were excluded.	Table 2 (narrative excluded): Chen [19], Cui [23], Fabbrini [25], Newsome [24] with reason for exclusion.
Study characteristics	17	Cite each included study and present its characteristics.	Section 3.2 and Table 1: 15 studies (n=1461 participants) detailed by design, country, sample, diversity indices, taxa.
Risk of bias in studies	18	Present assessments of risk of bias for each included study.	Section 3.2 summary and Supplementary Table S4 (NOS 6–9, $\kappa=0.85$).
Results of individual studies	19	For all outcomes, present, for each study: (a) summary statistics for each group (where appropriate) and (b) an effect estimate and its precision (e.g. confidence/credible interval), ideally using structured tables or plots.	Section 3.5.1–3.5.3; Tables 2–3; Supplementary Tables S2–S3; effect sizes with CIs.
Results of syntheses	20a	For each synthesis, briefly summarise the characteristics and risk of bias among contributing studies.	Section 3.5.1 and Discussion 4.4: Most high-quality NOS; moderate heterogeneity attributed to geography and antibiotic exposure.
	20b	Present results of all statistical syntheses conducted. If meta-analysis was done, present for each the summary estimate and its precision (e.g. confidence/credible interval) and measures of statistical heterogeneity. If comparing groups, describe the direction of the effect.	Figures 2–4; pooled SMD, logFC, OR/HR with CIs, I^2, τ^2 reported.
	20c	Present results of all investigations of possible causes of heterogeneity among study results.	Discussion 4.4.1: Explains moderate heterogeneity and consistency across continents.
	20d	Present results of all sensitivity analyses conducted to assess the robustness of the synthesized results.	Section 3.5.1 (leave-one-out) and 4.4.1 confirm unchanged directionality.
Reporting biases	21	Present assessments of risk of bias due to missing results (arising from reporting biases) for each synthesis assessed.	Section 3.3 and Supplementary Figure S1 (Egger's $p = 0.32$; no asymmetry).
Certainty of	22	Present assessments of certainty (or confidence) in the body of evidence for	Section 4.5 "Strength of Evidence"—

Section and Topic	Item #	Checklist item	Location where item is reported
evidence		each outcome assessed.	GRADE levels described.
**DISCUSSION**			
Discussion	23a	Provide a general interpretation of the results in the context of other evidence.	Section 4.1 "Comparison with Previous Meta-Analyses" (Cheng 2022, Reuben 2023, Li 2023).
	23b	Discuss any limitations of the evidence included in the review.	Section 4.4 "Strengths and Limitations": notes sample heterogeneity, antibiotic confounding, limited low-income regions.
	23c	Discuss any limitations of the review processes used.	Section 4.4.1: Acknowledges incomplete data reporting, lack of IPD.
	23d	Discuss implications of the results for practice, policy, and future research.	Sections 4.3 and 4.6: Implications for clinical microbiome monitoring and future meta-omics standardization.
**OTHER INFORMATION**			
Registration and protocol	24a	Provide registration information for the review, including register name and registration number, or state that the review was not registered.	Section 2 (Methods): PROSPERO registration CRD420251160970.
	24b	Indicate where the review protocol can be accessed, or state that a protocol was not prepared.	Section 2: Protocol prospectively registered; link provided in PROSPERO.
	24c	Describe and explain any amendments to information provided at registration or in the protocol.	Not applicable—no amendments post-registration.
Support	25	Describe sources of financial or non-financial support for the review, and the role of the funders or sponsors in the review.	Funding Statement: "This research was funded by the Victor Babeș University of Medicine and Pharmacy Timișoara." No funder involvement in analysis or writing.
Competing interests	26	Declare any competing interests of review authors.	Conflicts of Interest: "The authors declare no conflict of interest."
Availability of data, code and other materials	27	Report which of the following are publicly available and where they can be found: template data collection forms; data extracted from included studies; data used for all analyses; analytic code; any other materials used in the review.	Data Availability Statement: "All data are included in this article and supplementary materials; inquiries to corresponding author."
